# Characteristics and outcomes of percutaneous coronary interventions in patients with spontaneous coronary artery dissection. A study from the administrative minimum data set of the Spanish National Health System

**DOI:** 10.3389/fcvm.2022.1054413

**Published:** 2022-12-01

**Authors:** Fernando Alfonso, Cristina Fernández-Pérez, Náyade del Prado, Marcos García-Guimaraes, José Luis Bernal, Teresa Bastante, David del Val, María García-Márquez, Javier Elola

**Affiliations:** ^1^Department of Cardiology, Hospital Universitario de La Princesa, IIS-IP, CIBER-CV, Universidad Autónoma de Madrid, Madrid, Spain; ^2^Department of Preventive Medicine, Área Sanitaria de Santiago de Compostela y Barbanza, Instituto de Investigación de Santiago, Santiago de Compostela, Spain; ^3^Fundación Instituto para la Mejora de la Asistencia Sanitaria, Madrid, Spain; ^4^Department of Cardiology, Hospital del Mar – Parc de Salut Mar, Grupo de Investigación Biomédica en Enfermedades del Corazón, Institut Hospital del Mar d’Investigacions Mèdiques, Barcelona, Spain; ^5^Department of Information and Management Control, Hospital Universitario 12 de Octubre, Madrid, Spain

**Keywords:** acute myocardial infarction, acute coronary syndrome, angiography, coronary revascularization, complications, mortality, readmission, spontaneous coronary artery dissection

## Abstract

**Background:**

Coronary revascularization in patients with spontaneous coronary artery dissection (SCAD) is challenging. Indications and results of percutaneous coronary interventions (PCI) in SCAD patients are not well established.

**Aim:**

To assess indications and results of PCI in SCAD.

**Methods:**

The minimum basic data set of the Spanish National Health System (years 2016−2019) was used to identify 804 episodes of acute myocardial infarction (AMI) and SCAD, with a crude in-hospital mortality rate of 3%. Of these, 368 (46.8%) patients were revascularized with PCI during admission whereas 436 (54.2%) were managed conservatively.

**Results:**

Revascularization and in-hospital mortality rates both declined over the study period (p for trend both < 0.05). SCAD patients treated with PCI were older, more frequently male, and had higher frequency of diabetes, ST-segment elevation AMI and cardiogenic shock, compared to patients managed conservatively. The crude in-hospital mortality rate was higher in patients treated with PCI (4.9% vs. 1.4%; *p* = 0.004). However, after adjusting by propensity score (223 pairs) the in-hospital mortality rate was similar in the two groups (Adj OR: 1.21; 95%CI: 0.30−1.57; *p* = 0.76). Readmissions at 30-days were higher in patients managed conservatively (7.1 vs. 1.6%, *p* < 0.001) and this difference was maintained after propensity score adjustment (Adj average treatment effect: 2% vs. 12.2%; OR: 0.15; 95%CI: 0.04−0.45; *p* < 0.001).

**Conclusion:**

Revascularization is frequently used in unselected patients with AMI and SCAD but its use is declining. Patients with SCAD treated with PCI have a higher in-hospital mortality but this appears to be explained by their adverse baseline clinical characteristics.

## Introduction

Spontaneous coronary artery dissection (SCAD) is a rare but increasingly recognized cause of acute myocardial infarction (AMI) (1–4). This unique disease mainly affects middle-aged women and its most common clinical presentation is as acute coronary syndrome (ACS). The widespread use of urgent coronary angiography in patients presenting with ACS, including young females, the enhanced clinical awareness for the condition and the diagnostic insights provided by intracoronary imaging, explains the current improved diagnostic accuracy (1–4). Angiographically, SCAD may present with the classic double lumen pattern (dissection) but, more frequently, a characteristic image of diffuse or focal angiographic narrowing, caused by an intramural hematoma, is detected (1–4). SCAD is frequently associated with non-coronary vascular abnormalities (particularly fibromuscular dysplasia) and some recent studies even support a genetic association (1–4). In contradistinction to patients with AMI caused by an atherothrombotic lesion, in AMI secondary to SCAD a conservative medical treatment strategy is initially recommended (1–4). Prognosis is favorable in most patients with SCAD managed conservatively and late spontaneous healing of the vessel wall is part of the natural history of this condition. On the other hand, revascularization is challenging in this anatomic scenario due to the underlying fragile and already disrupted coronary vessel wall (1–4). Moreover, SCAD usually presents in tortuous small and distal vessel segments where interventions are technically complex. Percutaneous coronary interventions (PCI) in patients with SCAD are associated with a higher rate of procedural-related complications (including the extension of the dissection, intramural hematoma propagation and side-branch occlusion) and frequently obtain suboptimal angiographic results (1–4). Therefore, recent expert consensus documents from both sides of the Atlantic suggest reserving coronary revascularization for patients with ongoing or recurrent ischemia, those presenting with a complete vessel occlusion and for cases with high-risk anatomy (left main or proximal multivessel involvement) (1, 2). However, the available information on coronary revascularization in patients with AMI caused by SCAD remains scarce (1–7).

This study sought to assess the baseline characteristics and clinical outcomes (namely in-hospital mortality and 30-days readmissions for cardiovascular causes) of patients with SCAD undergoing coronary revascularization, using the registry of the minimum basic data set (MBDS) of the Spanish National Health System (NHS).

## Materials and methods

### Population

This is a retrospective observational study of all patients discharged from Spanish NHS hospitals with a diagnosis of AMI, during the period from 01 January 2016 to 31 December 2019. The source of the data was the MBDS of the NHS, using the coding of the International Classification of Diseases 10th Edition Clinical Modification (ICD-10-CM) (8).

The study population was divided into two groups: (1) patients with primary or secondary diagnosis of AMI (ICD-10 codes: I21.x) with primary or secondary diagnosis of SCAD (ICD-10 code: I25.42) –both diagnosis present at admission- *with* a PCI procedure (AMI-SCAD-PCI); and (2) patients with primary or secondary diagnoses of AMI (ICD-10 codes: I21.x) with primary or secondary diagnoses of SCAD (ICD-10 code: I25.42) –both diagnosis present at admission- *without* a PCI (AMI-SCAD-NPCI).

Multiple hospitalizations resulting from transfers between hospitals were considered as a single care episode. Episodes corresponding to patients under 18 years of age, those registered as voluntary discharges or with an unknown discharge outcome, episodes with stays of 1 day or less and discharged at home, were excluded. Moreover, to increase the diagnostic accuracy of SCAD, episodes with primary or secondary diagnosis of perforation or accidental injury during medical procedure; primary or secondary diagnosis of native or grafted coronary artery arteriosclerosis or transplanted heart disease, or chronic ischemic heart disease; secondary diagnostic of arteriosclerosis; or previous history of AMI, coronary angioplasty or aortocoronary revascularization surgery, were also excluded. ICD-10-CM codes used to identify exclusions are shown in Supplementary Table 1. Although episodes with previous stroke or AMI were excluded, the occurrence of stroke and AMI events after the diagnosis of SCAD was analyzed during follow-up.

### Statistical analysis

Categorical variables were expressed as number and percentage, while continuous variables were expressed as mean and standard deviation (SD) or median and interquartile range [25th−75th percentile] according to their distribution. Qualitative variables were analyzed using the chi-squared or the Fisher’s exact test and differences in continuous variables were compared using a 2-sided Student’s *t*-test or Mann-Whitney *U* test, according to their distribution for the unmatched comparison. Temporal trends were analyzed using a χ^2^ for trend.

Multilevel logistic regression models were specified and adjusted for the outcome’s variables analyzed: in-hospital mortality and 30 days readmissions for cardiovascular causes (Chapter 9, ICD 10), based on the methodology of the *Centers for Medicare and Medicaid Services* (CMS) for the AMI (9), adapted to the data structure of the MBDS, after grouping the secondary diagnoses according to the Condition Categories (10) updated yearly by the *Agency for Healthcare Research and Quality*. Variables included were those baseline characteristics found to be statistically significant on univariable analysis with an odds ratio (OR) > 1.00. Backward elimination regression was then performed with significance for inclusion being *p* < 0.05 and for elimination being ≥ 0.10. In-hospital mortality ratios were calculated from these specified models and the calibration was analyzed graphically after grouping patients in deciles with respect to the predicted probabilities, and tabulating the mean predicted vs. observed probabilities. Discrimination was assessed by the area under the receiver operating characteristic curves (AUROC). From the specified models, the ratios of in-hospital mortality (RSMR) were calculated (11). Cox model was used to adjust readmissions, and hazard ratio (HR) with CI 95% were calculated; to develop this model the variables included in the AMI 30-day readmissions adjusted risk model for by the CMS, were used (9). Assumption of proportionality was evaluated using tests the proportional-hazards assumption on the basis of Schoenfeld residuals after fitting a model.

To minimize potential selection biases in the comparison of outcomes between the two groups, the impact on in-hospital mortality and 30-day readmissions by cardiovascular causes was further assessed by matching propensity scores (option k-nearest neighbors matching, psmatch2, and Stata), selecting among the episodes with AMI-SCAD-PCI those with a profile more similar to each episode of AMI-SCAD-NPCI, according to the variables statistically significant in the risk-adjustment models. The matching was made from the risk adjustment models (9), with a 1:1 ratio and without replacement. The probability of in-hospital mortality, the effect of differences between groups (average treatment effect [ATT]) and OR with their 95% confidence intervals (95% CI), were calculated. Comparison of continuous and categorical variables between the matched groups were as previously stated for unmatched groups. A *p*-Value of < 0.05 was considered to be statistically significant. The graphical representation of the matching process was made using Kernel density plots. Statistical analyses were performed with STATA 16 and SPSS v21.0.

## Results

### In-hospital mortality

A total of 804 episodes of AMI-SCAD were identified after exclusions (Supplementary Figure 1), with a crude in-hospital mortality rate of 3% (Table 1). Of these, 436 (54.2%) episodes were AMI-SCAD-NPCI and 368 (45.8%) underwent PCI during the initial episode. Revascularization rates (51.5% −2016- to 40.2% −2019-) and in-hospital mortality (5.9% −2016- to 0.9% −2019-) significantly diminished over the study period (p for trend 0.029 and 0.01, respectively). Patients undergoing revascularization were older, more frequently male and smokers, and had higher frequency of diabetes, ST-segment elevation AMI, chronic obstructive pulmonary disease renal failure, heart failure and cardiogenic shock, compared to AMI-SCAD-NPCI patients. Fibromyalgia was more frequently recorded in AMI-SCAD-NPCI episodes (Table 1). Mean hospital stay was significantly longer in revascularized patients (7.7 vs. 6.8 days, *p* = 0.019). The crude in-hospital mortality rate was higher in AMI-SCAD-PCI patients compared with those with AMI-SCAD-NPCI (4.9% vs. 1.4%; *p* = 0.004).

**TABLE 1:** Differences between patients with spontaneous coronary artery dissection and acute myocardial infarction treated with and without percutaenous coronary intervention.

	Total	AMI-SCAD-NPCI	AMI-SCAD-PCI	*P*
			
	*N* = 804	*n* = 436	*n* = 368	
Age (years) (Mean ± Standard Deviation)	56.1 (12.4)	54.8 (11.2)	57.6 (13.6)	0.002
Sex (Female)%	67.7	86	45.9	<0.001
Fibromyalgia (M79.7) (%)	2.4	3.7	0.8	0.008
Smoking habit (Z72.0; F17.*) (%)	31.8	28.7	35.6	0.036
Dyslipidaemia (CC 25) (%)	35.4	33.5	37.8	0.206
Cardiogenic shock (R57.0) (%)	2.4	0.7	4.3	0.001
STEMI principal diagnosis (I21. *. except I21.A1 and I21.4)	60	51.8	69.6	<0.001
AMI complications (I23.0. I23.1. I23.2. I23.3. I23.6. I23.7. I23.8. I24.1) (%)	2	1.4	2.7	0.175
Metastatic cancer, acute leukemia and other severe cancers (CC 8−9) (%)	0.7	0.9	0.5	0.539
Diabetes mellitus (DM) or DM complications except proliferative retinopathy (CC 17−19. 123) (%)	11.6	6.4	17.7	<0.001
Protein-calorie malnutrition (CC 21) (%)	0.2	0	0.5	0.123
Chronic liver disease (CC 27−29) (%)	1.1	0.7	1.6	0.206
Dementia or other specified brain disorders (CC 51−53) (%)	0.2	0	0.5	0.123
Major psychiatric disorders (CC 57−59) (%)	0.2	0.2	0.3	0.904
Other cardio-respiratory failure and shock (CC 84 except 785.51) (%)	2.9	2.3	3.5	0.294
Congestive heart failure (CC 85) (%)	7.2	4.4	10.6	0.001
Valvular and rheumatic heart disease (CC 91) (%)	9.2	8.7	9.8	0.602
Hypertension (CC 95) (%)	36.3	38.1	34.2	0.26
Stroke (CC 99−100) (CC 99−100) (%)	0.2	0	0.5	0.123
Cerebrovascular disease (CC 101−102, 105) (%)	0.6	0.5	0.8	0.522
Vascular disease and complications (CC 106−108) (%)	0.1	0.2	0	0.358
Chronic obstructive pulmonary disease (COPD) (CC 111) (%)	3	1.4	4.9	0.004
Pneumonia (CC 114−116) (%)	0.7	0.2	1.4	0.064
Renal failure (CC 135−140) (%)	2.9	1.4	4.6	0.006
Trauma; other injuries (CC 166−168, 170−174) (%)	1.7	0.9	2.7	0.052
Crude in-hospital mortality rate (%)	3	1.4	4.9	0.004
30-days readmissions (circulatory diseases)	4.6	7.1	1.6	<0.001

AMI, acute myocardial infarction.

CC, condition categories (9).

**FIGURE 1:**
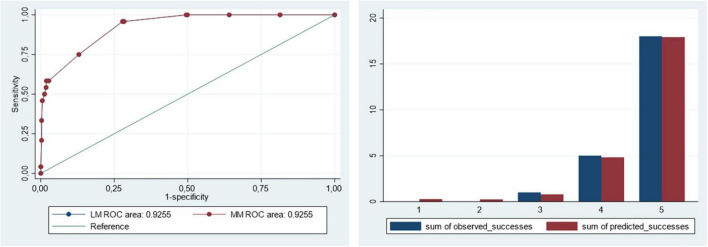
Discrimination and calibration of the logistic and multilevel risk adjustment models for in-hospital mortality.

The multilevel logistic regression model for in-hospital mortality did not show an effect at hospital level (median OR: 1). The logistic regression model for in-hospital mortality showed a good discrimination (AUROC: 0.926; 95%CI 0.883−0.968; *p* < 0.001) and calibration (Figure 1 and Table 2). Age > 55 years, ST-segment elevation AMI, AMI complications, cardiogenic shock and chronic pulmonary disease were independent predictors of in-hospital mortality. The presence of cardiogenic shock at admission was the variable with the highest OR for in-hospital mortality (OR: 127.4; 95%CI 25.1−646.6; *p* < 0.001). Coronary revascularization did not have a statistically significant effect on in-hospital mortality when was tested in the logistic regression model (OR: 1.49; 95%CI 0.45−4.9; *p* = 0.513).

**TABLE 2:** Multilevel logistic regression model of risk adjustment for in-hospital mortality.

Mortality	Odds ratio	*P*	95% conf. interval	
Age > 55 years	23.22	<0.001	3.61	149.48
STEMI	11.46	0.02	1.50	71.78
AMI complications (I23.0. I23.1. I23.2. I23.3. I23.6. I23.7. I23.8. I24.1)	10.37	0.02	1.50	90.07
Cardiogenic shock	127.39	<0.001	25.09	646.64
Chronic obstructive pulmonary disease	10.46	<0.001	2.93	38.68

STEMI, ST elevation myocardial infarction; AMI, acute myocardial infarction.

CC, condition categories (9).

**FIGURE 2:**
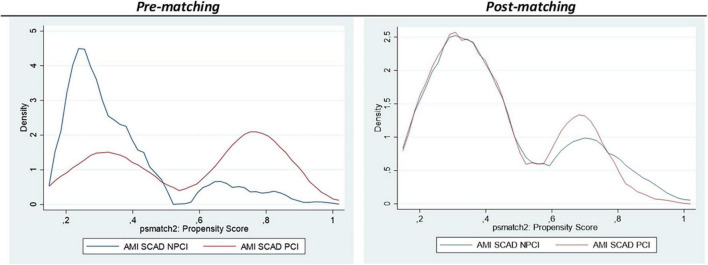
Kernel Density plots representing the pre and post matching. In-hospital mortality.

**FIGURE 3:**
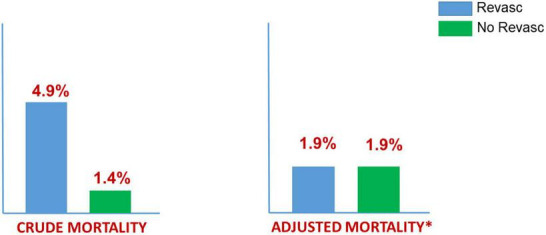
In-hospital mortality: SCAD (2016–2019) MBDS (ICD-10-CM) Spanish NHS. 368 (45%) Revascularization vs. 436 (56%) No Revascularization. 223 “pairs” adjusted by propensity score (*). Patients undergoing Revascularization: older, males, diabetes, STEMI, and shock.

In addition, propensity score matching resulted in 223 pairs. Figure 2 depicts the Kernel density plots before and after matching, and Table 3 the patients’; characteristics. After matching, in-hospital mortality was similar in both groups (ATT: 2.69 vs. 2.24; OR: 1.21; 95%CI: 0.30−1.57; *p* = 0.760). Mortality results are summarized in Figure 3.

**TABLE 3:** Baseline characteristics of the matched cohorts.

	AMI-SCAD-PCI 223	AMI- SCAD-NPCI 223	p1-p2	p1(1-p1) + p2 (1-p2)	SDM
Male	24.7	27.4	−0.027	0.459	−0.059
Age > 55 years	48.1	52.6	−0.045	0.556	−0.081
STEMI	26	28.2	−0.022	0.468	−0.047
AMI complications (I23.0. I23.1. I23.2. I23.3. I23.6. I23.7. I23.8. I24.1)	40.1	37.5	0.026	0.536	0.049
Cardiogenic shock	0	1	−0.010	0.000	Undefined
Chronic obstructive pulmonary disease	1.9	1.9	0.000	0.137	0.000

In-hospital mortality. STEMI, ST elevation myocardial infarction; AMI, acute myocardial infarction; SDM, standard deviation of the mean.

CC, condition categories (9).

### 30-days readmissions from cardiovascular diseases

**FIGURE 4:**
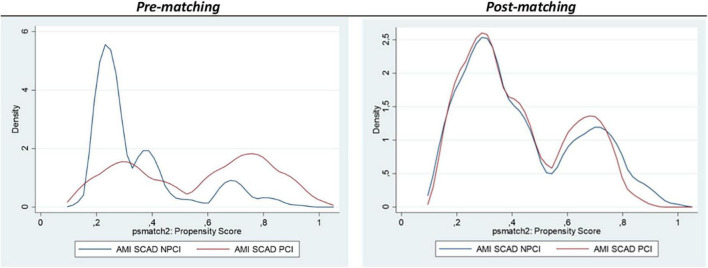
Kernel Density plots representing the pre- and post- matching. 30-days readmissions.

**FIGURE 5:**
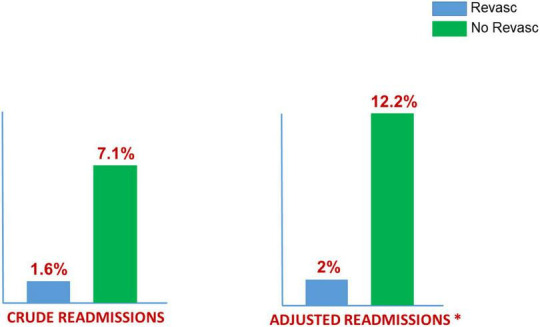
30-days readmissions: SCAD (2016–2019) MBDS (ICD-10-CM) Spanish NHH. 370 (45%) Revasculariation vs. 436 (56%) No Revascularization. 196 “pairs” adjusted by propensity score (*).

The crude readmission rate was 1.6% in AMI-SCAD-PCI patients compared with 7.1% in AMI-SCAD-NPCI (*p* < 0.001). In addition, readmissions due to AMI were also more frequent among AMI-SCAD-NPCI patients (2.5% vs. 0%, *p* = 0.003). Cox regression model for 30-days readmissions by cardiovascular causes showed a fair discrimination (Harrell’s C: 0.683; 95%CI 0.512−0.743) (Supplementary Table 2). Female gender was associated with a higher readmission rate (HR: 4.75; 95%CI: 1.59−14.24; *p* = 0.001). The adjusted hazard ratio for cardiovascular readmissions in AMI-SCAD-PCI patients was also lower than in AMI-SCAD-NPCI patients (Adj HR: 0.30; CI95% 0.11−0.80; *p* = 0.017). Propensity score matching for 30-days cardiovascular readmissions resulted in 196 pairs (Table 4 depicts patients’ characteristics; Figure 4, Kernel density plots before and after matching). After matching, 30-days cardiovascular readmissions were 2.0% in AMI-SCAD-PCI and 12.2% in AMI-SCAD-NPCI (ATT: 2.0 vs. 12.2; OR: 0.15; 95%CI: 0.04−0.45; *p* = 0.001). Readmission results are summarized in Figure 5.

**TABLE 4:** Baseline characteristics of the matched cohorts.

	AMI-SCAD-PCI *N* = 196	AMI-SCAD-NPCI *N* = 196	p1-p2	p1(1-p1) + p2(1-p2)	SDM
Male	24.5	26.5	−0.020	0.457	−0.044
Age > 55 years	41.3	43.4	−0.021	0.541	−0.039
AMI complications (I23.0. I23.1. I23.2. I23.3. I23.6. I23.7. I23.8. I24.1)	27	30	−0.030	0.475	−0.063
Severe infection; other infectious diseases (CC 1, 3−7)	2	1.5	0.005	0.141	0.036
Metastatic cancer and acute leukemia (CC 8)	0.5	0.5	0.000	0.071	0.000
Diabetes mellitus (DM) or DM complications except proliferative retinopathy (CC 17−19, 123)	9.7	9.7	0.000	0.303	0.000
Iron deficiency or other/unspecified Anemia and blood disease (CC 49)	6.6	5.6	0.010	0.252	0.040
Congestive heart failure (CC 85)	6.1	6.1	0.000	0.243	0.000
Valvular and rheumatic heart disease (CC 91)	10.2	4.6	0.056	0.306	0.183
Specified arrhythmias and other heart rhythm disorders (CC 96−97)	12.8	11.7	0.011	0.344	0.032
Renal failure (CC 135−140)	2	2	0.000	0.141	0.000

30-day readmissions. STEMI, ST elevation myocardial infarction; AMI, acute myocardial infarction; SDM, standard deviation of the mean.

CC, condition categories (9).

## Discussion

The main findings of this large nationwide study on SCAD are the following: (1) Coronary revascularization is still frequently performed in patients with AMI caused by SCAD; (2) Revascularization rates diminished overtime during the study period; (3) SCAD patients undergoing revascularization during hospital admission present with adverse clinical characteristics as compared with those managed conservatively; (4) In-hospital mortality is three-fold higher in patients requiring revascularization, as compared with those managed with medical therapy alone, but after adjusting for the adverse baseline characteristics of revascularized patients, the differences in mortality were no longer statistically significant; (5) Recurrent hospitalization for cardiovascular causes is higher in SCAD patients managed conservatively than in those requiring coronary revascularization and this difference persists after adjusting for potential confounders. Recurrent AMI at 30-days was also more frequently seen in patients treated conservatively.

### General considerations of coronary revascularization in spontaneous coronary artery dissection

A careful assessment of the clinical presentation and angiographic findings is critical in the clinical decision-making process of coronary revascularization in SCAD. Consensus documents on this entity emphasize that clinical presentation mandates PCI in selected high-risk patients to improve myocardial perfusion and reduce myocardial damage (1–7). Patients with SCAD suffering active/ongoing or recurrent ischemia and those with totally occluded coronary arteries and a large area of jeopardized myocardium should be considered for prompt coronary revascularization (1–7). In addition, patients with hemodynamic/arrhythmic instability and those with cardiogenic shock should also be considered for revascularization. Revascularization should be avoided in most patients stabilized after the acute ischemic insult, in asymptomatic patients with a TIMI 3 coronary flow and in those at low risk, including patients with distal lesions or small vessels (1–7). A relative prolonged hospitalization has been recommended for patients with SCAD (1, 2). An early discharge of patients managed conservatively might help to explain the relatively high rate of recurrent hospitalization seen in some of these patients in our study. Of interest, recent studies suggest that the use of dual antiplatelet therapy may increase the risk of adverse events in SCAD patients managed conservatively (12).

Revascularization of patients with SCAD is challenging and associated with a relatively high rate of procedural-related complications (1–7). These include iatrogenic dissection, advancement of the guidewire into the false lumen, dissection extension, hematoma propagation, abrupt vessel closure and unplanned requirement of multiple stents. PCI is frequently associated with suboptimal results (residual lesions caused by residual dissection or intramural hematoma) although, in most cases, coronary flow is improved after the procedure. In this anatomic scenario the aim is to restore normal coronary flow rather than obtaining an optimal angiographic vessel wall appearance (1–7). At long-term, patients treated with PCI may suffer from in-stent restenosis or stent thrombosis but also from SCAD recurrences at different coronary segments. Coronary surgery may be preferred to PCI in patients with left main disease and those with proximal multivessel involvement. Coronary surgery may be also required as a bailout procedure in patients experiencing a PCI-related complication. Although early results of surgery are usually favorable, graft occlusion is frequently found at follow-up due to competitive flow caused by healing of the native vessel (1–7).

### Previous studies on coronary revascularization in spontaneous coronary artery dissection

There are no randomized clinical trials comparing coronary revascularization vs. a conservative medical management in SCAD. Accordingly, the management of these patients is primarily based on observational studies and experts’ opinion (1–7). Currently, a conservative medical management is recommended as the first-line strategy for these patients (1, 2).

(a) *Observational studies*: Most observational studies assessing results of coronary revascularization in SCAD are small and confounded by selection bias and lack of long-term follow-up and, therefore, the results should be interpreted with major caution. In a classical retrospective study from the Mayo Clinic, Tweet et al. (13) analyzed the results of coronary revascularization in SCAD patients. Revascularization was performed in 95 out of 189 patients (50%), with a procedural failure rate of 53%. Up to 10% of patients in the conservative group experienced early SCAD progression requiring revascularization. Although baseline characteristics were similar in both groups, revascularized patients more frequently presented with occluded vessels. The 5-year rates of target vessel revascularization and recurrent SCAD were no different in the revascularization vs. conservative group. Hassan et al. (14), from the Vancouver group, compared results of 75 SCAD patients treated with PCI with 328 SCAD patients managed conservatively. All patients undergoing PCI presented with AMI and the procedure was considered successful in 34.7%, partially successful in 37.3%, and unsuccessful in 28%. Adverse event rates were more frequent in the PCI group, both in-hospital (29.3% vs. 2.8%, *p* < 0.001), and at a median follow-up of 3.7 years (58.7% vs. 22.6%, *p* < 0.001). Interestingly, the need for repeated revascularization was higher in the PCI group. However, the results were not corrected for the higher-risk characteristics of patients requiring revascularization. Recently, Kotecha et al. (15) compared 215 SCAD patients undergoing revascularization, from 3 European national cohort studies, with a matched cohort of 221 patients conservatively managed. SCAD patients undergoing PCI were at higher risk at presentation (including ST-segment elevation AMI, proximal location and total vessel occlusion). PCI complications occurred in 38.6% of cases (13% serious), but coronary flow could be improved in 84% of cases initially presenting with an abnormal flow. Clinical outcome and left ventricular function were favorable and similar in patients with and without revascularization. Garcia-Guimaraes et al. (16, 17) compared outcomes according to the revascularization status in the prospective Spanish SCAD Registry. Of 389 SCAD patients 84 underwent PCI as the initial strategy. Patients initially treated with PCI presented more frequently with ST-segment elevation AMI, proximal disease and occluded vessels. Procedural success, according to a predefined flow criterion, was obtained in 84% of cases. Despite the higher-risk clinical profile of patients with initial revascularization and a high rate of PCI-related complications, long-term clinical results (median 29 months) were similar in patients with and without revascularization (16).

Finally, although in our study PCI was more frequently used in men, most previous studies have found a similar rate of coronary revascularization in women and men with SCAD (18–21). A recent study from Vancouver (21) analyzed the characteristics of men with SCAD. In this study men had more frequently a physical trigger and less frequently an emotional trigger than women but the need for revascularization and the results of the interventions were similar in both genders. Of interest, in that study readmission for chest pain was more frequent in women than in men (21).

(b) *Meta-analyses:* Meta-analyses are an attractive tool to evaluate all the available evidence, particularly when pooling of small observational studies is required because large studies are unavailable. In an early meta-analysis including 440 SCAD patients Shamloo et al. (22) found that 21% of those initially treated conservatively required coronary revascularization due to recurrent myocardial ischemia. Martins et al. (23) performed a meta-analysis including 11 studies and 631 SCAD patients (253 treated with PCI or surgery) with no difference in mortality, myocardial infarction, or SCAD recurrence between patients treated with revascularization or managed conservatively. However, revascularization was associated with a higher risk of repeat revascularization during follow-up. Finally, in a more recent and comprehensive meta-analysis, Bocchino et al. (24) included 24 observational studies with 1,720 SCAD patients. After a mean clinical follow-up of 28 ± 14 months, a conservative approach was associated with lower rate of target vessel revascularization without differences in death, AMI, heart failure or SCAD recurrences.

(c) *Administrative studies:* To the best of our knowledge, the present study is the first analysis comparing the results of coronary revascularization in unselected SCAD patients using a large nationwide administrative dataset. Most previous studies on SCAD using administrative databases have not focused on revascularization results. A recent study using the Nationwide Readmissions Database (years 2010−2015) analyzed SCAD patients according to revascularization status, but only in women ≤ 60 years presenting with AMI (25). This registry includes ∼50% of all hospital admissions in the United States. The revascularization group (*n* = 1,273, 68%), as compared with the conservative therapy group (*n* = 600, 30%), had more frequently STEMI and cardiogenic shock. Admission to teaching hospitals was associated with conservative therapy. Propensity-score matched analyses (546 pairs) found no significant difference in in-hospital death, 30-day readmission, and recurrent AMI, between the strategies. However, the reason to perform revascularization in up to 2/3 of the SCAD patients in this study, was not clarified. In our study, we did not exclude patients by age or gender. Moreover, the Spanish NHS covers virtually all the population with a medical emergency (including AMI). In addition, most of our patients were treated conservatively, according to recent recommendations. Whether this difference in the revascularization rates is a result of a more recent analysis (years 2016−2019 in our series) or a more conservative approach to SCAD in Europe, as compared with the United States, remains unclear. Interestingly, we detected a declining use of revascularization during the study period. Although one might attribute the declining mortality seen in more recent years to a more frequent use of conservative medical management, this assumption remains speculative, and only should be considered as hypothesis generationg, because this relationship cannot be demonstrated from our data. Furthermore, we used a very strict and restrictive methodology to identify SCAD patients and also well-established criteria (RSMR and propensity score analyses) to adjust for the differences between patients with and without revascularization. Our results, demonstrating that the worse prognosis of SCAD patients requiring revascularization is a consequence of their adverse clinical characteristics, are consistent with and complement previous findings from clinical registries on this condition (1–7, 13–17). Moreover, the lower risk of readmission for cardiovascular causes, and recurrent AMI, seen in patients undergoing revascularization is of major clinical interest, but requires confirmation in additional studies.

### Limitations

Some study limitations should be acknowledged. An inherent limitation of any study on AMI based on administrative datasets is the lack of the required clinical and anatomic granularity that impedes a precise characterization of the patients’ profile. The incidence of SCAD is low but variable in the different previous studies in relation to the definition used and the type of study (2, 26–29). In addition, the use of filters in administrative studies, to refine the diagnosis of SCAD and prevent misdiagnosis of patients with atherosclerotic MI (i.e., prior MI, prior stroke), may introduce a selection bias in the study population. Likewise, the percentage of female patients in our study, and in all studies based on administrative databases, is lower than in clinical studies (18–21, 26–29). However, only administrative databases provide the sample size needed to ascertain clinical outcomes in relatively rare causes of AMI, including SCAD. The use of the MBDS of the Spanish NHS ensures uniform codification criteria and our methodology to select and compare AMI patients has been previously demonstrated to be reliable and accurate (30). However, the precise clinical indication for revascularization, the type of intervention selected and detailed data on procedural results or complications, were not available. In addition, we lumped together revascularization procedures performed at diagnosis with those eventually required during hospitalization after an initial conservative strategy. Moreover, the potential implications of angiographic patterns suggesting intramural hematomas (Type 2−3) (31) could not be assessed as this variable was not available in our study. Finally, our clinical follow-up was limited to the study of readmissions at 30 days. A longer clinical follow-up is indeed required to ascertain the clinical implications of revascularization in SCAD.

## Conclusion

Coronary revascularization is still frequently used in unselected patients with SCAD presenting with AMI although its use is declining in recent years. These patients have adverse clinical characteristics as compared with patients managed medically which appear to explain their higher in-hospital mortality. However, readmissions for cardiac causes at 1 month are reduced in SCAD patients undergoing revascularization during the index hospitalization.

### Impact on daily practice

Spontaneous coronary artery dissection patients requiring revascularization have adverse clinical characteristics that explain their higher hospital mortality. Revascularization should not be withhold in SCAD when clinically indicated.

## Data availability statement

The datasets presented in this article come from a nationwide administrative public registry. Requests to access the datasets should be directed to FA, falf@hotmail.com.

## Ethics statement

Ethical review and approval was not required for the study on human participants in accordance with the local legislation and institutional requirements. Written informed consent for participation was not required for this study in accordance with the national legislation and the institutional requirements.

## Author contributions

All authors contributed to the design, analysis, drafting, and approved the final version.

## Abbreviations

AMI: acute myocardial infarction
ACS: acute coronary syndrome
MBDS: minimum basic data set
NHS: National Health System
PCI: percutaneous coronary intervention
SCAD: spontaneous coronary artery dissection.
