# Supplementary material for: Characteristics and outcomes of percutaneous coronary interventions in patients with spontaneous coronary artery dissection. A study from the administrative minimum data set of the Spanish National Health System
Source: Front Cardiovasc Med. 2022 Dec 1;9:1054413. doi: 10.3389/fcvm.2022.1054413 (PMC9754633; doi:10.3389/fcvm.2022.1054413)
Supplement: Supplementary file 1 [file Data_Sheet_1.docx]

**Supplementary Material**

**Supplementary Table 1. ICD-10 Codes used for exclusion criteria**

|  | **ICD 10 Codes** |
| --- | --- |
| Atherosclerosis of coronary artery bypass graft (s) and coronary artery of transplanted heart with angina pectoris | I25.7* I25.81* I25.700 I25.701 I25.708 I25.709 I25.710 I25.711 I25.718 I25.719 I25.720 I25.721 I25.728 I25.729 I25.730 I25.731 I25.738 I25.739 I25.750 I25.751 I25.758 I25.759 I25.760 I25.761 I25.768 I25.769 I25.790 I25.791 I25.798 I25.799 I25.810 I25.811 I25.812 |
| Accidental puncture and laceration of a circulatory system organ or structure during a procedure | I97.5* |
| Percutaneous coronary interventionism or coronarography | 02703ZZ 02704ZZ 02713ZZ 02714ZZ 02723ZZ 02724ZZ 02733ZZ 02734ZZ 02C03ZZ 02C04ZZ 02C13ZZ 02C14ZZ 02C23ZZ 02C24ZZ 02C33ZZ 02C34ZZ B2000ZZ B2001ZZ B200YZZ B2010ZZ B2011ZZ B201YZZ B2020ZZ B2021ZZ B202YZZ B2030ZZ B2031ZZ B203YZZ B2100ZZ B2101ZZ B210YZZ B2110ZZ B2111ZZ B211YZZ B2120ZZ B2121ZZ B212YZZ B2130ZZ B2131ZZ B213YZZ 0270346 0270356 0270366 0270376 0270446 0270456 0270466 0270476 0271346 0271356 0271366 0271376 0271446 0271456 0271466 0271476 0272346 0272356 0272366 0272376 0272446 0272456 0272466 0272476 0273346 0273356 0273366 0273376 0273446 0273456 0273466 0273476 02703E6 02704E6 02713E6 02714E6 02723E6 02724E6 02733E6 02734E6 027034Z 027035Z 027036Z 027037Z 02703D6 02703DZ 02703EZ 02703F6 02703FZ 02703G6 02703GZ 02703Z6 02703ZZ 027044Z 027045Z 027046Z 027047Z 02704D6 02704DZ 02704EZ 02704F6 02704FZ 02704G6 02704GZ 02704Z6 02704ZZ 027134Z 027135Z 027136Z 027137Z 02713D6 02713DZ 02713EZ 02713F6 02713FZ 02713G6 02713GZ 02713Z6 02713ZZ 027144Z 027145Z 027146Z 027147Z 02714D6 02714DZ 02714EZ 02714F6 02714FZ 02714G6 02714GZ 02714Z6 02714ZZ 027234Z 027235Z 027236Z 027237Z 02723D6 02723DZ 02723EZ 02723F6 02723FZ 02723G6 02723GZ 02723Z6 02723ZZ 027244Z 027245Z 027246Z 027247Z 02724D6 02724DZ 02724EZ 02724F6 02724FZ 02724G6 02724GZ 02724Z6 02724ZZ 027334Z 027335Z 027336Z 027337Z 02733D6 02733DZ 02733EZ 02733F6 02733FZ 02733G6 02733GZ 02733Z6 02733ZZ 027344Z 027345Z 027346Z 027347Z 02734D6 02734DZ 02734EZ 02734F6 02734FZ 02734G6 02734GZ 02734Z6 |
| Chronic vascular disease | E08.51 E09.51 E10.51 E11.51 E13.51 I70.0 I70.1 I70.201 I70.202 I70.203 I70.208 I70.209 I70.211 I70.212 I70.213 I70.218 I70.219 I70.221 I70.222 I70.223 I70.228 I70.229 I70.291 I70.292 I70.293 I70.298 I70.299 I70.301 I70.302 I70.303 I70.308 I70.309 I70.311 I70.312 I70.313 I70.318 I70.319 I70.321 I70.322 I70.323 I70.328 I70.329 I70.391 I70.392 I70.393 I70.398 I70.399 I70.401 I70.402 I70.403 I70.408 I70.409 I70.411 I70.412 I70.413 I70.418 I70.419 I70.421 I70.422 I70.423 I70.428 I70.429 I70.491 I70.492 I70.493 I70.498 I70.499 I70.501 I70.502 I70.503 I70.508 I70.509 I70.511 I70.512 I70.513 I70.518 I70.519 I70.521 I70.522 I70.523 I70.528 I70.529 I70.591 I70.592 I70.593 I70.598 I70.599 I70.601 I70.602 I70.603 I70.608 I70.609 I70.611 I70.612 I70.613 I70.618 I70.619 I70.621 I70.622 I70.623 I70.628 I70.629 I70.691 I70.692 I70.693 I70.698 I70.699 I70.701 I70.702 I70.703 I70.708 I70.709 I70.711 I70.712 I70.713 I70.718 I70.719 I70.721 I70.722 I70.723 I70.728 I70.729 I70.791 I70.792 I70.793 I70.798 I70.799 I70.92 I71.2 I71.4 I71.6 I71.9 I72.0 I72.1 I72.2 I72.3 I72.4 I72.5 I72.6 I72.8 I72.9 I73.1 I73.81 I73.89 I73.9 I77.0 I77.1 I77.2 I77.3 I77.4 I77.5 I77.6 I77.810 I77.811 I77.812 I77.819 I77.89 I77.9 I78.0 I79.0 I79.1 I79.8 I80.10 I80.11 I80.12 I80.13 I80.201 I80.202 I80.203 I80.209 I80.211 I80.212 I80.213 I80.219 I80.221 I80.222 I80.223 I80.229 I80.231 I80.232 I80.233 I80.239 I80.291 I80.292 I80.293 I80.299 I82.0 I82.210 I82.211 I82.220 I82.221 I82.290 I82.291 I82.3 I82.401 I82.402 I82.403 I82.409 I82.411 I82.412 I82.413 I82.419 I82.421 I82.422 I82.423 I82.429 I82.431 I82.432 I82.433 I82.439 I82.441 I82.442 I82.443 I82.449 I82.491 I82.492 I82.493 I82.499 I82.4Y1 I82.4Y2 I82.4Y3 I82.4Y9 I82.4Z1 I82.4Z2 I82.4Z3 I82.4Z9 I82.501 I82.502 I82.503 I82.509 I82.511 I82.512 I82.513 I82.519 I82.521 I82.522 I82.523 I82.529 I82.531 I82.532 I82.533 I82.539 I82.541 I82.542 I82.543 I82.549 I82.591 I82.592 I82.593 I82.599 I82.5Y1 I82.5Y2 I82.5Y3 I82.5Y9 I82.5Z1 I82.5Z2 I82.5Z3 I82.5Z9 I82.621 I82.622 I82.623 I82.629 I82.721 I82.722 I82.723 I82.729 I82.A11 I82.A12 I82.A13 I82.A19 I82.A21 I82.A22 I82.A23 I82.A29 I82.B11 I82.B12 I82.B13 I82.B19 I82.B21 I82.B22 I82.B23 I82.B29 I82.C11 I82.C12 I82.C13 I82.C19 I82.C21 I82.C22 I82.C23 I82.C29 K55.1 K55.8 K55.9 M31.8 M31.9 |
| AMI history | I25.2 |
| Stroke history | I69.* |
| Percutaneous coronary interventionism (PCI) | 027*3**, 027*4** |
| Percutaneous coronary interventionism history | Z95.5, Z98.61 or any PCI code in the previous year |
| CABG | 0210*, 0211*, 0212*, 0213* |
| CABG history | Z95.1; T82.211D, T82.211S, T82.212D, T82.212S, T82.213D, T82.213S, T82.218D, T82.218S or any CABG code during the previous year. |
| CABG: coronary artery bypass grafting | |

**Supplementary Figure 1. Chart-flow of study population exclusions**

368

182.685

**AMI SCAD**

≤ 1 day of stay and discharge to home alive

Discharge to other hospital and outcome unknown

Voluntary discharge

Unknown discharge destination

**Total exclusions**

**< 18 years old**

1.025

9

22

2

11

**Exclusions**

221

804

1

Chronic vascular disease

AMI history

Stroke history

Previous PCI history

Accidental puncture and laceration of a circulatory system organ or structure during a procedure

13

51

9

76

17

Previous CABG history

7

46

Percutaneous coronary interventionism or coronariography not performed

Atherosclerosis of coronary artery bypass graft (s) and coronary artery of transplanted heart with angina pectoris

1

**AMI**

CABG+PCI episode

2

**AMI-SCAD-PCI**

**AMI-SCAD-NPCI**

436

**Supplementary Table 2. Cox regression model of risk adjustment for 30-days readmissions**

|  | **HR** | **P** | **95% confidence intervals** | |
| --- | --- | --- | --- | --- |
| Age > 55 years | 0.72 | 0.38 | 0.34 | 1.51 |
| Female | 4.75 | 0.01 | 1.59 | 14.23 |
| AMI | 1.17 | 0.73 | 0.48 | 2.87 |
| STEMI | 1.41 | 0.45 | 0.58 | 3.40 |
| Heart Failure (CC 85) | 0.50 | 0.51 | 0.07 | 3.90 |
| Angina (CC 88) | 2.13 | 0.46 | 0.29 | 15.45 |
| Valvular and rheumatic heart disease (CC 91) | 0.72 | 0.65 | 0.17 | 2.99 |
| Vascular or circulatory disease (CC 106-109) | 1.43 | 0.73 | 0.19 | 10.70 |
| Asthma (CC 113) | 2.12 | 0.23 | 0.62 | 7.27 |
| AMI. Acute myocardial infarction; STEMI: ST elevation myocardial infarction. CC: Condition Categories (Pope et al)^9^ | | | | |
